# The Impact of Temporal Changes in Irradiated nMAG Polymer Gels on Their Applicability in Small Field Dosimetry in Radiotherapy

**DOI:** 10.3390/gels8100629

**Published:** 2022-10-04

**Authors:** Aurimas Krauleidis, Diana Adliene, Zivile Rutkuniene

**Affiliations:** Physics Department, Kaunas University of Technology, Studentu Str. 50, 51368 Kaunas, Lithuania

**Keywords:** polymer gels, radiotherapy, small field dosimetry, dose profiles

## Abstract

As advanced radiotherapy techniques progress to deliver a high absorbed dose to the target volume while minimizing the dose to normal tissues using intensity-modulated beams, arcs or stereotactic radiosurgery, new challenges occur to assure that the high treatment dose is delivered homogeneously to the tumor. Small irradiation field sizes (≤1 cm^2^) that tightly conform to precise target regions and allow for the deliverance of doses with a high therapeutic ratio, are of particular interest. However, the small field dosimetry using conventional dosimeters is limited by the relative large size of the detector. Radiation-sensitive polymer gels have the potential to meet this dosimetry challenge due to their almost unlimited ability in resolving three-dimensional dose distributions of any shape and makes them unique and suitable for the evaluation of dose profiles and the verification of complex doses. In this work, dose distributions in nMAG gels that have been irradiated to different doses by applying a 6 MV FFF photon beam collimated to 1 cm^2^, were analyzed and the dose profiles were evaluated by applying a gamma passing rate criteria of 3%/3 mm and considering different post-irradiation time intervals between the irradiation and the gels read out process. X-ray CT and NMR imaging procedures were used for the dose evaluation. It was found that the shape and uniformity of the dose profiles were changing due to post-irradiation polymerization and gelation processes, indicating time dependent growing uniformity which was better expressed for the higher delivered doses. It was estimated that in order to obtain acceptably symmetric small field dose profiles, a longer post-irradiation time is needed for getting the full scope of the polymerization as compared with the recently recommended 24 h period between irradiation and the read out processes of the dose gels. An estimated overall uncertainty (double standard deviation, 95% confidence level) of 3.66% was achieved by applying R2 measurements (NMR read out), and a 3.81–applying X-ray CT read out for 12 Gy irradiated gels 56 h post-irradiation. An increasing tendency for the uncertainty was observed with a decreasing post-irradiation time. A gamma passing rate of 90.3% was estimated for the 12 Gy irradiated gels and, 56 h post-irradiation, the X-ray CT evaluated gels as well as a gamma passing rate of 92.7% was obtained for the NMR evaluated gels applying a 3%/3 mm passing criteria.

## 1. Introduction

During the last decade, gel dosimetry in modern radiotherapy was found to be one of the most attractive applications of polymer gels. Modern advanced radiation therapy is represented by intensity-modulated radiation therapy (IMRT), volumetric modulated arc therapy (VMAT) or stereotactic radiotherapy (SRT) which allow for a highly conformal 3D delivery of high radiation doses to a well-defined target volume with a nominal spatial accuracy of a few mm adapted to the planning target volume (PTV) [1,2]. Dose distributions in small irradiation field (≤1 cm^2^) applications when the field size tightly conforms to precise target regions and allows for the delivering of doses with a high therapeutic ratio are of particular interest. The development of all of these radiotherapy methods is aimed to deliver the requested dose to the target volume trying to spare healthy tissues or organs at risk for ensuring a good quality of life [3]. Increasing treatment complexity requires the precise verification of treatment dose distribution plans that are prepared using complex computer algorithms aimed at the modelling of the irradiation system. Dose verification with the results of experimental measurements performed using different dosimeters is an important part of the treatment planning process as it provides an independent confirmation that the planned treatment dose will be accurately delivered to the target. To verify the accuracy of the IMRT, VMAT and SRT techniques, it is advantageous to measure the radiation dose distribution in three dimensions. Conventional dosimeters such as ionization chambers, thermoluminescent detectors (TLDs) and radiochromic film are effective at quantifying the radiation dose, but are limited to 1D or 2D measurements [4]. The small field dosimetry using conventional dosimeters is additionally limited due to the relatively large size of these detectors [5]. Radiation-sensitive polymer gels, whose response to irradiation is based on the radiation-induced polymerization of a monomer (and often co-monomer) species suspended in a gelatin matrix, have the potential to meet these limitations and can be used for the recording of dose profiles and the verification of complicated dose distributions in external beam therapy [6]. Various techniques, such as magnetic resonance imaging (MRI) [7,8], X-ray computed tomography (CT) [9,10], optical computed tomography (OCT) [9,10,11,12,13] or ultrasound [14,15] can be used for the evaluation of 3D dose distribution changes caused by the polymerization of irradiated polymer gels. Raman spectroscopy may also be considered as a power full tool for investigating radiation-induced changes in polymerized gel dosimeters, since the radiation-induced changes of the vibrational bands of corresponding monomers/polymers in gels correlate with the absorbed dose. This method has the potential to provide dose distributions with a very high (~1 µm) spatial resolution, but may be limited by the achievable penetration depth of light into the dosimeter [16,17]. Taking into account that the spatial resolution of gels is unlimited and depends on the imaging equipment capabilities only, optical CT and MRI read out methods seem to be most suitable for the read out of irradiated dose gels. The advantages of an optical CT system to evaluate polymer gel dosimeters include their low noise and also low SNR as compared to MRI images; the equipment is simple and low cost, and may produce 3D dose maps with suffcient spatial resolution, accuracy and precision. It was demonstrated that using optical CT to evaluate polymer gel dosimeters for the verification of complex radiotherapy treatment plans 3D dose maps could lead to them being produced within an hour with a sub-milimeter spatial resolution, 3% accuracy and less that 1% precision [13].

*Dose gels and their applications in external beam radiotherapy.* Despite the large variety of polymer gels used for dosimetry applications [18,19,20,21,22], all of them contain radiation-sensitive monomers which tend to form polymer networks after irradiation, gelatin (scaffold) and water (>80%, usually). Due to the large amount of water in these type of hydrogels, water radiolysis causes a primary response in dose [20]. First of all, due to ionizing radiation interaction with water, electrons (e^−^), positively charged water ions (H_2_O^+^) and water molecules in an excited state (H_2_O*) are formed. Excited water molecules may decompose to hydrogen and hydroxyl radicals (H_2_O* → H + OH). The resultant electrons lose their energy and become hydrated (e^−^ + nH_2_O → e^−^_aq_). Hydronium ions and very reactive hydroxyl radicals are formed due to the reaction of the positively charged water ions with water (H_2_O^+^ + H_2_O → H_3_O^+^ + OH) and the excited water molecules separate to hydrogen and hydroxyl radicals (H_2_O*→H + OH). Reacting together hydrated electrons, hydronium ions, hydrogen and hydroxyl radicals are forming molecular species of water, molecular hydrogen and hydrogen peroxide (H_2_O_2_). Thus, the basic principle of gel dosimetry is the reaction of free radicals and molecular species with the active material (monomer) and formation of dose dependent polymer structures. [20].

Being nearly tissue equivalent, polymer gels are suitable and unique alternatives compared with conventional dosimeters due to their ability to resolve three-dimensional (3D) dose distributions in an irradiated volume of any shape with high accuracy and precision. The main advantages of gel dosimeters over other dosimetry techniques are: the ability to score a dose in three dimensions, the ability to integrate a dose without perturbing the radiation beam and producing integrated dose maps [11] and the ability to act as a phantom, allowing for the accurate modelling of radiation dose distributions. The possibility of a visible verification of the radiation-induced polymerization result in irradiated gel dosimeters is another great advantage.

However, it should be noted that there are a number of complications associated with gel dosimetry, such as: the diffusion of the active material, the application of toxic materials (Acrylamide), the oxygen sensitivity of polymer gels causing inconsistencies in their radiation sensitivity and other problems that remain to be addressed [19,23]. The most important disadvantage is that the dose response of gels is inhibited by the presence of oxygen. It has been reported that the oxygen contained in the polymer gel may reduce the performance by removing the free radicals produced by radiation [24]. To solve this problem and to produce dose gels that can work under normal atmospheric conditions, normoxic gel dosimeters were developed, adding a small amount of an antioxidant (such as ascorbic acid, gallic acid, trolox, (hydroxymethyl) phosphonium chloride, THPC and others) to the main formulation of a polymer gel in order to bound oxygen into metallo-organic complexes. In this way, bound oxygen is prevented from binding the free radical and, hence, the polymerization reactions essential for polymer gel dosimetry are not inhibited.

The advantages of admixing an oxygen scavenger to polymer gels have been thoroughly investigated during the last decades [25,26]. The majority of performed studies have investigated the dose response of the irradiated gels (MAG, PAG, VIPET and others) containing tetrakis (hydroxymethyl) phosphonium chloride (THPC), which was added as an antioxidant to various gel dosimeter formulations, but also other chemicals as oxygen scavengers have been suggested. Interesting results were found in [26] investigating basic dosimetric properties: linear dose range, sensitivity, precision, accuracy and the dose rate dependence of the methacrylic acid-based gels (MAGADIT) where the role of dithiothreitol as an oxygen scavenger was discussed. These gels demonstrated a linear dose response up to a dose range of a minimum of 24 Gy. The highest sensitivity of 0.54 Gy^−1^ s^−1^) was observed in the polymer gel having lowest scavenger concentration (2 mmol/kg) [26]. This paper also discussed the applicability of methacrylic acid based gels as multidimensional dose detector in small field radiotherapy in the case when 10 MV flattening filter-free (FFF) beam was used for irradiation. The obtained results were similar to those found performing measurements with reference radiochromic films. However, it was pointed out that dose distribution maps are particularly sensitive to the high dose gradients due to possible special errors that may contribute to discrepancies between measured and planned dose distributions. This led to the conclusion that MAGADIT gels are not suitable for the low dose rate range applications, thus not applicable for the recording of the complex high gradient radiation fields with locally strongly varying dose rates. This implies that the three-dimensional verification of intensity-modulated radiotherapy dose distributions, especially in small field dosimetry, is still a challenge even for 3D gel dosimetry, since the high dose gradients must be analyzed. It should be noted that, in the past, the majority of papers discussing dose gel applications were related to the quantification of doses delivered to the target, dose calibration and evaluation of 3D dose distributions in the irradiated volume [19,27,28], however, with the development of modern radiation treatment technologies, the small field dosimetry, which requires a high dose delivery accuracy, became one of the hottest topics where gel dosimeters could be applied [29,30,31,32].

The absorbed dose value and exact delivery location are two main parameters defining the successful deliverance of the treatment dose to the target. A comparison of spatial dose distributions (dose profiles) in irradiated gels with the treatment dose plans provided by a standard treatment planning system, TPS, can be obtained through the use of Low’s gamma function, which quantifies the agreement of the combined metrics of the dose difference and distance-to-agreement between two dose distributions into a single ‘gamma’ value [33,34,35].

Applying gamma evaluation method which has been proposed by Low, D.A [33,34], it is possible to evaluate dose distribution quantitatively using two dosimetry parameters: distance to agreement, DTA, (indicates misalignment) and percent dose difference, DD, (indicates discrepancy). DTA is the nearest distance between the point of reference dose and the same dose on the examined dose distribution. The dose discrepancy is calculated at the same point assuming that the alignment between two dose distributions is perfect. The simultaneous evaluation of both parameters allows for calculation of “gamma index”as it is shown in the following equation [34]:

(1) $$\Gamma \left(\vec{r_{e}},\vec{r_{r}}\right)=\sqrt{\frac{{\left|\vec{r_{e}}-\vec{r_{r}}\right|}^{2}}{\Delta d^{2}}+\frac{{\left[D_{e}\left(\vec{r_{e}}\right)-D_{r}\left(\vec{r_{r}}\right)\right]}^{2}}{\Delta D^{2}}}$$

where $\vec{r_{e}}$ and $\vec{r_{r}}$ are the vector positions of the evaluated and reference points, respectively; *D_e_*($\vec{r_{e}}$) and *D_r_*($\vec{r_{r}}$) are the calculated and reference doses, respectively; and Δ*d* and Δ*D* are the DTA and DD criteria, respectively. A 3% dose and 3 mm distance between points are usually used for the verification of dose treatment plans calculated by TPS by means of experimentally measured dose distribution data.

The generalized Γ function can be computed for any pair $\vec{r_{e}}$ and $\vec{r_{r}}$. Therefore, for each reference point, there are as many values of Γ as there are evaluated points. The minimum value of Γ is the value of *γ* [34]:

(2) $$\gamma \left(\vec{r_{r}}\right)=min\left\{\Gamma \left(\vec{r_{e}},\vec{r_{r}}\right)\right\}\forall \left\{\vec{r_{e}}\right\}$$

The results of the LINAC beam profile measurements (part of RT QA procedure) for small field (diameter of 5 and 10 mm) irradiation using VIPAR polymer gel, radiographic films and a PinPoint ionization chamber [36] indicated a relatively good agreement between the TPS calculated and the polymer gel measured data. The penumbras measured with the polymer gel were smaller than the penumbras measured with a film or the PinPoint chamber. The relative depth dose measurements showed a good agreement between the film and gel. A potential of gel dosimetry in small field applications was also shown in [37] where the results of the gamma knife facility beam profile measurements in small irradiation fields (diameter of 4, 8 and 16 mm) using an nPAG-based dosimeter and Gafchromic films were presented. It was concluded that relatively accurate beam profile data can be obtained using dose gels for small field dosimetry. Another group of authors [28] analyzed dose profiles and have reported that the dose distribution in an irradiated polymer gel phantom showed a 97% gamma passing rate using 3%/2mm criteria, which is applicable in RT QA. The application of an NMPA dosimeter, and BANG-GEL-QA™ for dose verification in cyber knife radiotherapy, were discussed in [12]. It was shown, that the measured dose distributions of NMPA gel and the commercial BANG-GEL-QA™ polymer gel agreed with TPS calculated dose distributions very well indicating gamma passing rates that were higher than 95% for the selected 5%/2 mm criteria. Collimated beam diameter was at least 32 mm in this experiment

As was already mentioned above, the spatial resolution of gels is unlimited, but it strongly depends on the parameters of read out techniques such as MRI, X-ray computed tomography and optical CT, that are used to quantify radiation-induced chemical changes in polymer gels and acquire the spatial dose distribution.

The successful application of imaging modalities (MRI and OCT) for the evaluation of polymer gel dosimeters applicability in the small field dosimetry are provided in studies [38,39], where it was shown that the normoxic NIPAM polymer gel dosimeters can be applied for the assessment of small field dose deliveries using both read out techniques: cone beam optical computed tomography and MRI. Both methods indicated an acceptable gamma passing rate (<1) since a voxel agreement of 95% for the target level was achieved between the treatment plan and gel-measured dose distributions, applying 3% dose and 3 mm distance-to-agreement gamma function criteria. Interesting results on the X-ray CT application for the dose response evaluation in polymer gel is provided in paper [10]. In this study, the nMAG gel dosimeter and the home-made cylindrical phantoms were used for the CT dose and image quality assurance. It was shown that the radiation dose differences between dose; the CT evaluated nMAG gel dosimeter and the 10 cm ionization chamber were less than 1%.

Despite an impressive number of papers that focused on the investigation of dose gels and their applicability for dosimetry purposes [18], there was not much attention paid to the investigation of the temporal behavior of gel dosimeters after irradiation, just roughly assuming that gels must be read out not any earlier than 12 h after irradiation. However, gelation and polymerization reactions in polymer gels can continue for many hours after irradiation, causing temporal evolution/instability in dose responses [12,39,40,41], thus influencing the adequacy of the results of the dose profile measurements.

Due to the fact that the long term polymerization dynamic and the gelation processes in irradiated dose gels depends on various external and internal factors and remains still an opened question, temporary changes of measured dose profiles in small beam field irradiated nMAG gels were evaluated using an X-ray CT and MRI read out and analyzed against gamma index criteria.

## 2. Results and Discussion

**Figure 1:**
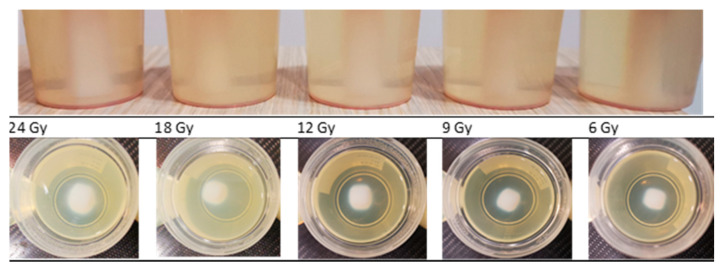
Photographs of nMAG gels irradiated with various doses applying small field beam irradiation geometry (photographs were taken just after the irradiation).

Before starting a detailed analysis of the dose profiles, a visual inspection of the dose gel samples which were irradiated by applying small field external beam (1 cm^2^) geometry has been performed. The polymerized volume can be easily recognized from the photographs of the gels irradiated to various doses using a 6 MeV photon beam (Figure 1), where the density differences between the not irradiated and polymerized part of the gels are seen. However, it is also clearly seen that the margins of the irradiated volume (especially in the transversal plane) are not sharp enough and the blurring increases with the increased delivered dose. These discrepancies between planned and polymerized volume may occur due to specific gel polymerization features and a possible diffusion of the polymer segments. Dose delivery uncertainties can also contribute to the formation of the polymerized volume.

It should be noted that in order to assess the temporal dose gel performance, the samples were evaluated just after irradiation, after 56 h, 240 h and 2300 h. An evaluation of the gels 2300 h post-irradiation was performed in order to check the long-term stability of the polymerized volume.

### 2.1. Evaluation of Dose Gels Performance Due to Irradiation Using X-ray CT

It is known [42] that a dose evaluation in irradiated gels may be performed using X-ray CT scans for the reconstruction of linear attenuation data related to the polymerization-caused optical density changes. Unlike MRI, the image acquisition method with X-ray CT is less sensitive to temperature changes and has the advantages of a lower cost and rapid image acquisition time. Easy accessibility is another advantage because CT devices are present in most clinics. Nevertheless, the process of polymerization has only a small effect on the attenuation coefficient of the gel and the corresponding signal to noise ratios (SNRs) in images is low. Additionally, CT examination contributes to the dose in the gel, thus requesting a more detailed analysis of the polymerization processes in irradiated gels.

**Figure 2:**
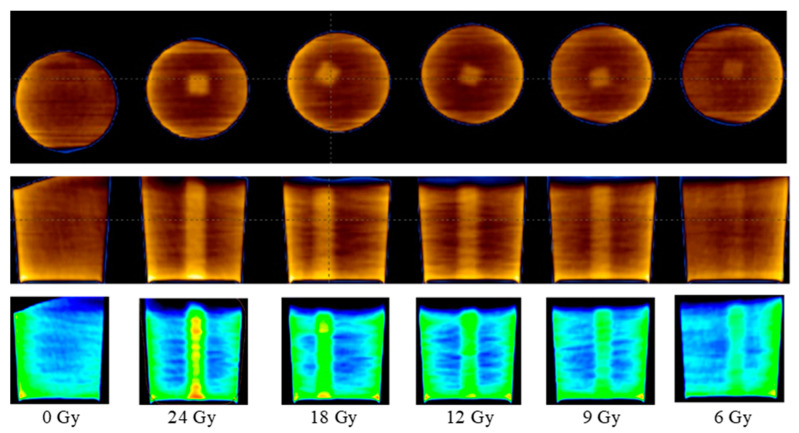
CT scans of irradiated polymer gels samples without and with colored indication of optical density changes (bottom row).

CT scans were used to assess the temporal dose gel performance after irradiation and to evaluate the dose distribution profiles in gels irradiated with different doses. Figure 2 shows CT images of gel samples obtained 56 h after the irradiation. A colored indication of optical density changes in the polymerized volume was provided in order to follow the increasing tendency of the density due to the polymerization of gels with the increasing irradiation dose.

A ore detailed analysis of density variations related to the dose distribution in irradiated gel vials was performed by measuring the CT number at the central position of the transverse plane situated at a 2.5 cm depth from the bottom side where it crosses with the symmetry axis of the irradiated volume, since the delivery of 100% of the dose was planned in this point. Density variations in the irradiated gels were obtained by analyzing CT numbers (Hounsfield units) in the CT scans.

As mentioned above, X-ray CT can be used as a scanning technique of polymer gel dosimeters since CT scans provide pixel-based digital images of the scanned objects. Digital images include information on the linear attenuation coefficient values of each transverse plane pixel, depending on the density changes of the irradiated gels due to radiation-induced processes. The radiation-induced linear attenuation coefficient changes are mainly attributed to the electron density changes originating from the expulsion of water in the polymer. The CT number (*N_CT_*) provided in Hounsfield units (HU) is the main parameter for the CT image evaluation [19]. *N_CT_* is a measure of the linear attenuation coefficient of the sample (*μ*) relative to that of water (*μ_w_*):

(3) $$N_{CT}=\frac{\mu -\mu w}{\mu w}\times 1000$$

The CT number depends on the atomic number of the investigated media and its density, also the quality of the beam which is used for sample scanning is important. In HU scale, air has the value of—1000 HU, water—0 HU. Hounsfield unit values for bones or metal objects may vary from several hundred to several thousand HU.

Since the linear attenuation coefficient (and therefore CT number) is affected by the density, changes in the irradiated gel CT number (Δ*N_CT_*) are directly proportional to a changes in gel density (Δ*ρ_gel_*) [11,19]:

(4) $$\Delta \rho_{gel}=KN_{CT}$$

where *K* is a function of not-irradiated gel density. For the nMAG gel, *K* ≈ 1, thus the Δ*N_CT_* expressed in HU is numerically equivalent to the gel density change in kg·m^−^^3^ [11].

**Figure 3:**
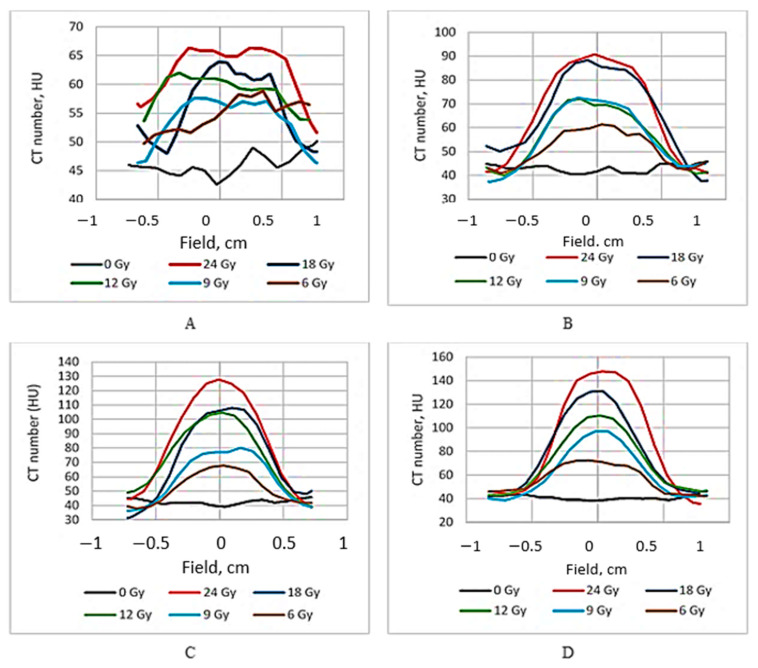
Dose profiles of irradiated nMAG gels CT evaluated at different post-irradiation times: (**A**) just after irradiation; (**B**) after 56 h; (**C**) after 240 h; and (**D**) after 2300 h.

The dose distribution profiles of the irradiated gels based on the CT number evaluation are provided in Figure 3.

**Figure 4:**
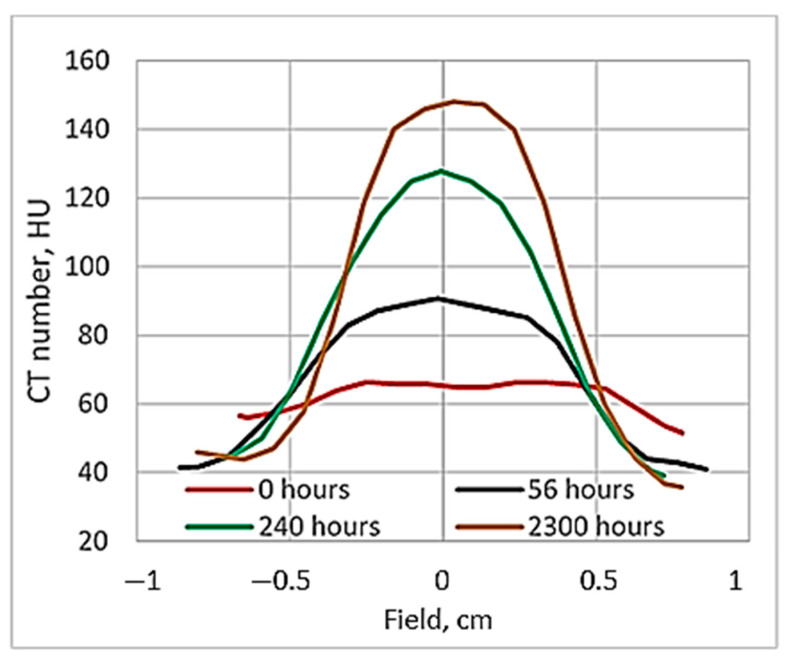
Comparison of 24 Gy irradiated gel dose profiles evaluated at different post-irradiation times.

It is known [19] that the ideal dose distribution in the irradiated target should follow the Gaussian shape if the flattering filter-free beam is applied for the irradiation. However, it is evident (Figure 3) that this was not the case when evaluating the gels just after the irradiation, since the radiation-induced polymerization processes were still active and additional time was needed to reach a thermodynamic equilibrium of the irradiated system. The dose profiles of gels which were evaluated after the certain post irradiation time are showing a tendency of becoming more uniform and symmetric. This tendency is better expressed for high dose irradiated gels. The flatness of the dose profiles was decreasing with the increasing post-irradiation time (especially for high dose irradiated gels) (Figure 4), indicating density changes in the irradiated volume related to post-irradiative polymerization and gelation processes within this volume.

**Figure 5:**
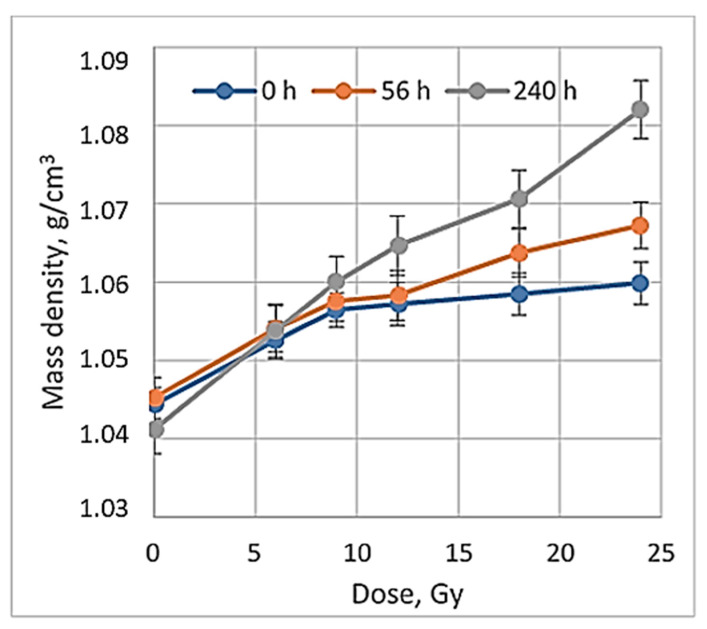
Post-irradiation time depending mass density changes in irradiated gels.

When exploring the numerical equivalency of HU to mass density changes (Equation (4)), the dose sensitivity of irradiated gels was evaluated. For this purpose, CT number values were calculated from the scans of irradiated gels applying the CT calibration curve, which was developed and used in the TPS system to match the mass density values obtained from the CT images with the data provided in the TPS system. A dose sensitivity of 1.76 ± 0.11 HU/Gy was estimated for the irradiated nMAG gels in the dose range up to 12 Gy (Figure 5). 

Similar sensitivity results for the MAGAT gels were reported in [42], where mass density changes in irradiated polymer gels were also discussed. Since it is evident that no additional mass is added to polymer gels through irradiation, observed mass density changes may result from the mass redistribution within the polymer system, or from gels volume change due to irradiation, which is linked with the potential loss of spatial integrity in polymer gels [19]. Density changes of the irradiated dose gels may occur due to the presence of two parallel processes running in irradiated gels: gelation and post-irradiation polymerization During the gelation process, there is a transition from a liquid to a semi-liquid phase due to the formation of macromolecular structures from a branched polymer structure [43]. It is important to note that this process is extremely fast when the solution is cooled down below 35 °C, but the evolution is much slower in later phases. This process can take up to 30 daysor longer and may cause shrinkage of the gel by losing water, which leads to the density changes in irradiated gels contributing to the evolution/instability in the dose responses [10,39,43,44,45]. Post-irradiation polymerization reactions progress up to 10 h after the irradiation [19,42], however, they do not fully stop due to the presence of remaining long-term radicals. In order to control the influence of the temporal dose response variations on the final result, the reading of the dosimetric information is usually recommended to be performed at least 24 h after irradiation, assuming that this time period is sufficient enough to achieve the chemical equilibrium of the system [7,12]. A 24 h interval between the irradiation and evaluation of the polymer gels was suggested due to the lack of detailed information regarding the time-dependent post-irradiation polymerization and gelation processes in gels, which are limited by the complexity of the required measurement methods and complicated analysis of the obtained results [44].

**Figure 6:**
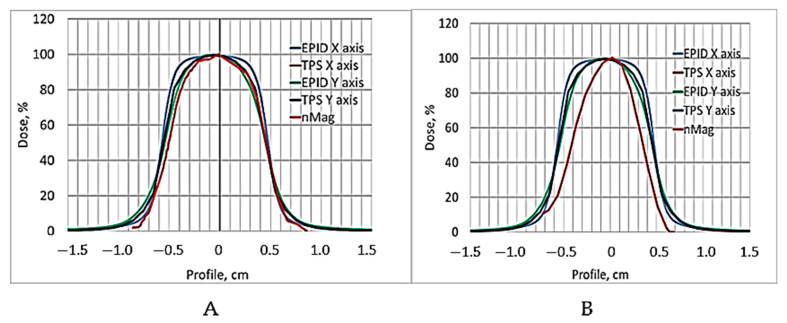
12 Gy dose profiles used for comparison: (**A**) TPS, EPID and CT evaluated dose profile of irradiated nMAG gel obtained 56 h post-irradiation, (**B**) TPS, EPID and CT evaluated dose profile of irradiated nMAG gel obtained 240 h post-irradiation. Gels dose profiles are indicated in red.

It seems that 24 h of post-irradiation time can be sufficient for the gels’ calibration when the whole volume of the sample is irradiated with the same dose, however, according to our results, more time between the irradiation and CT evaluation is needed to get reliable results applicable for small field dosimetry. This hypothesis was tested by comparing the dose profiles calculated by the treatment planning system, electronic portal imaging data (EPID), which is commonly used for dose verification, and the CT evaluation-based experimental data of irradiated gels. The comparison of dose distributions provided by TPS with the dose distributions obtained from the detector measurement data was performed using gamma function analysis [33]. The dose profiles of gels irradiated with 12 Gy 56 h post-irradiation and after 240 h were selected for investigation. The investigated dose profiles are provided in Figure 6.

Already, visual observation has shown that the gels dose profile data evaluated 240 h post-irradiation drastically differ from the values suggested by the TPS, thus indicating that the applicability of these data in small field dosimetry is not possible. A ore detailed quantitative evaluation of the dose distributions obtained using a polymer dosimeter was based on a gamma passing rate estimation [33], applying 3%/3 mm evaluation criteria. The results on the quantitative comparison (shape similarity with gamma distribution) of the TPS dose profiles calculated for the small field treatment (1 cm^2^) with the EPID-measured dose profile data and with X-ray CT evaluated dose distribution values in the 12 Gy irradiated gel 56 h post-irradiation, are provided in Table 1.

The estimated gamma passing rate for the EPID measurements was 95.6% and for the nMAG gel 90.3%. In general, the passing rate for the 12 Gy irradiated nMAG gel evaluated 56 h post-irradiation met the requirement (*γ* < 1), however, for a highly precise and accurate dosimetry, the gamma index (passing rate) should be around at least 97%. These findings led to the conclusion that the dose evaluation time of 56 h after the irradiation of the gels was not optimal and should be adjusted.

### 2.2. Evaluation of Dose Response of Irradiated Gels Using MRI

MRI is currently known to be the most accurate methods for gel dosimeter evaluation and one of the most appropriate evaluation methods for displaying the three-dimensional dose distribution with an advanced spatial resolution. In monomer/polymer gel dosimetry, the conversion of co-monomers to polymer aggregates, upon irradiation, alters the mobility of the surrounding water molecules [45]. This results in a change in the spin–spin relaxation rate R2 (=1/T2). Both the transverse relaxation time (T2) or the spin–spin relaxation rate (R2) can be used to perform a dose evaluation.

**Figure 7:**
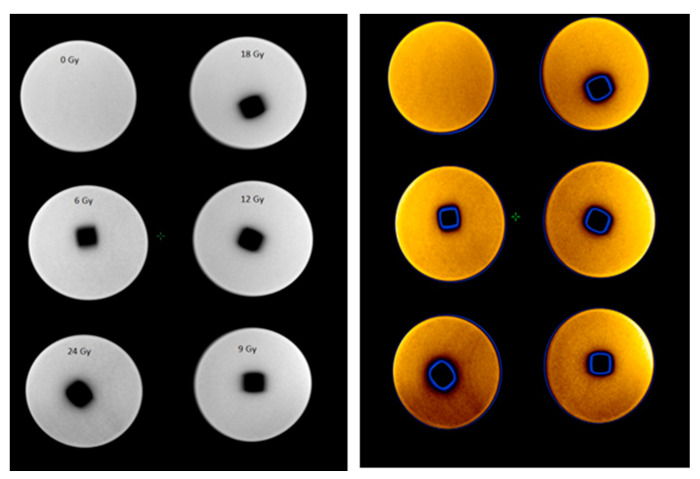
NMR images of polymer gel samples irradiated to different doses.

The NMR images (R2 maps) of polymer gels obtained 56 h post-irradiation are provided in Figure 7. (Colored indication of polymerized areas on the central transversal plane are provided for visibility).

**Figure 8:**
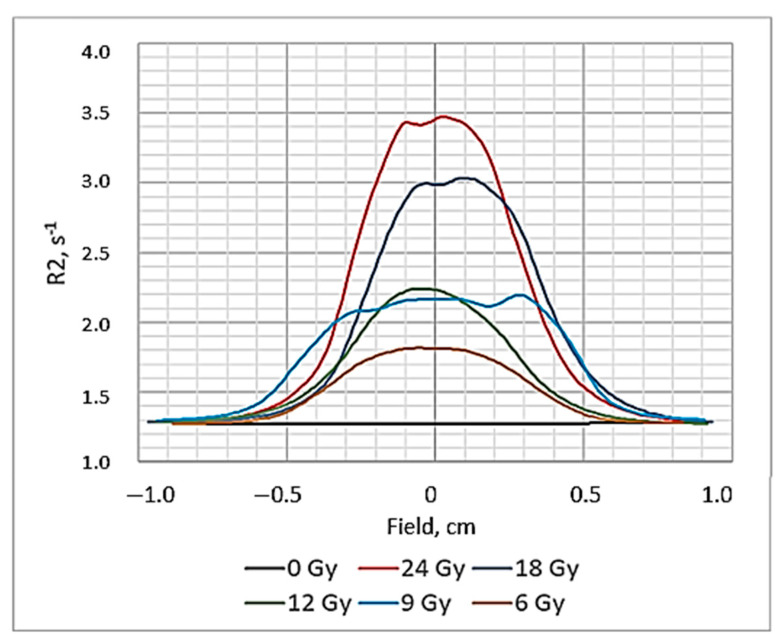
Dose profiles of irradiated gels evaluated by MRI 56 h post-irradiation.

**Figure 9:**
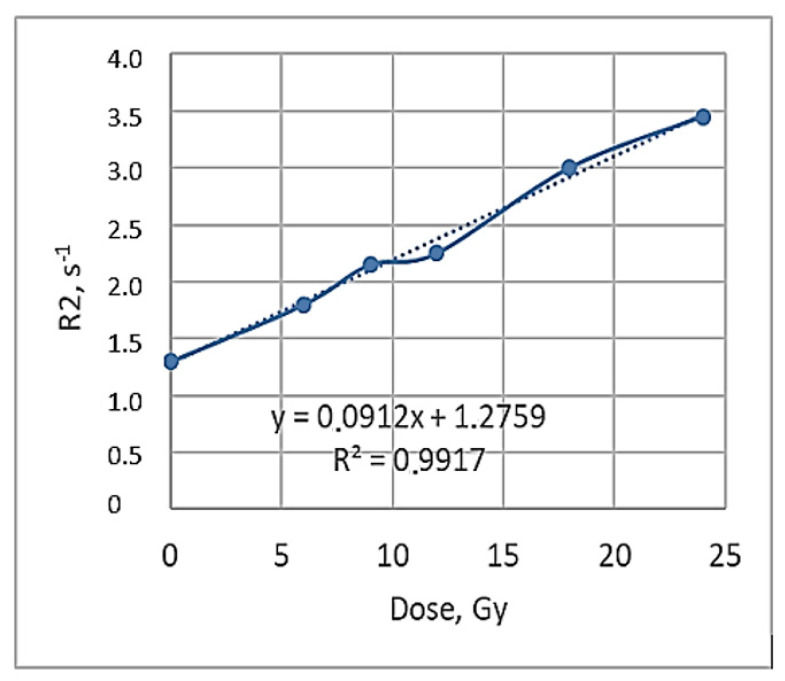
Relaxation rate R2-related dose sensitivity curve of irradiated gels evaluated by MRI 56 h post-irradiation.

Obtained R2 maps recording the dose distribution in the polymerized area of the irradiated gels were used for the calculation of the relaxation rate R2 values and the construction of dose profiles. In parallel, R2-related dose response curves were also derived, of which the slope represents the R2-dose sensitivity of the gel dosimeter and is used for a quantitative comparison between the different gel formulations and MRI imaging techniques [46]. The dose profiles of the irradiated gels obtained 56 h post-irradiation are shown in Figure 8 and the R2-dose sensitivity curve of the irradiated gels evaluated by MRI 56 h post-irradiation is shown in Figure 9. A dose sensitivity of 0.09 s^−1^Gy^−1^ was estimated for the analyzed nMAg gels.

**Figure 10:**
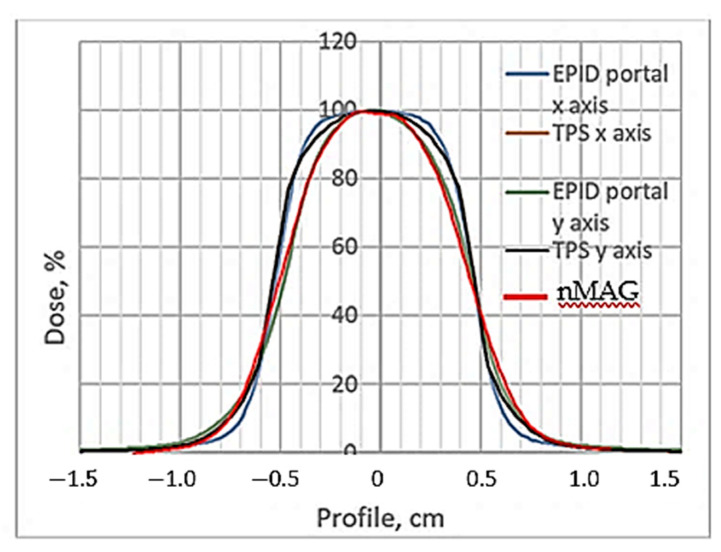
TPS, EPID and NMR evaluated dose profile of 12 Gy irradiated nMAG gel obtained 56 h post-irradiation. NMR evaluated gel’s dose profile is indicated in red.

For the quantitative evaluation of the dose distributions in the irradiated polymer dosimeter, a gamma passing rate estimation of 3%/3 mm, after applying the evaluation criteria, was applied. The TPS, EPID dose profiles and R2-dose response curve for the 12 Gy irradiated gel dosimeter are shown in Figure 10 and the results of the quantitative comparison (shape similarity with gamma distribution) of the TPS dose profiles calculated for the small field treatment (1 cm^2^) with EPID-measured dose profile data and with the R2-dose response curve data (NMR evaluation), are provided in Table 2. The NMR read out of the gels was conducted 56 h post-irradiation.

The shapes of the dose profiles of the irradiated gels obtained from the NMR images were similar to those obtained after the performed X-ray CT was read out. A comparison of MRI dose profiles measured 56 h post-irradiation with the TPS virtually calculated profiles indicated a gamma passing rate of 92.7% for the MRI read out (3%/3 mm criteria). It was slightly higher, compared to the CT evaluation, but still had not exceeded 97%, which is recommended when performing precise and accurate dosimetry procedures, thus indicating only a modest potential for the application of investigated nMAG dose gels in small field radiation dosimetry in radiotherapy. A relatively small gamma passing rate could be explained by the fact that the MRI imaging process is influenced by several factors, including inconsistencies in temperature, the inhomogeneity of the magnetic field, the presence of eddy currents and also the temporal stability of irradiated gels [47]. When analyzing the dose R2 response as a function of time, two different kinds of instability in monomer/polymer dosimeters can be distinguished [48,49]: (1) post-irradiation polymerization reactions that are retarded by the encapsulation of reactive sites on the polymer aggregates and are limited to the first 12 h after irradiation, and (2) the gelation process in gelatin-based dose gels which proceeds 50 h after manufacture and continues for more than a month. The mechanism responsible for this instability is the formation of macromolecular collagen structures that provoke a change in the local mobility of water molecules. In order to obtain more precise measurement results, molecular kinetics in irradiated gelatin gels should be considered and enough time should be allocated prior to the start dose evaluation. The allocated time should be longer than the now recommended 24 h post-irradiation.

### 2.3. Comparison of Dose Profiles in Irradiated Polymer Gels Obtained Using CT and NMR Read Out

There is a lack of detectors having parameters required in small field dosimetry: high signal to noise ratio, high spatial resolution, low direction dependence, low energy dependence, high stability, biological tissue equivalency. In this paper, we are discussing mainly two parameters of polymer gel detectors: high spatial resolution and accurate dose delivery in high gradient fields. As it was already mentioned earlier in this paper the spatial resolution of dose gels is unlimited, but it strongly depends on the parameters of the read out techniques that are used for the quantification of radiation-induced chemical changes: the polymerization and gelation of irradiated gel dosimeters.

Voxel-related spatial resolution of the submillimeter range was achieved in the case of CT-based gels evaluation and 0.5 × 0.5 × 5 mm voxels were used in the case of NMR-based gels evaluation.

Comparisons of the experimental dose profiles obtained from the EPID and gel dosimetry measurements with dose profiles provided by TPS (Figure 6 for CT and Figure 10 for NMR) revealed that the most differences were observed in the penumbra and out of field regions. Since an accurate beam profile is one of the required parameters for TPS, the inacuracies in measurements play an important role. In order to assess the impact of penumbra on dose delivery accuracy, penumbra and full width at half maximum (FWHM) values measured in 1 cm^2^ field are provided in Table 3. The penumbra was calculated between 80% and 20% of the central axis dose on both left and right sides. The results are presented for 12 Gy dose delivery, taking into account that gels were evaluated 56 h post-irradiation.

In a comparison between the TPS and polymer gels profiles, no significant dose differences were observed in the case of CT evaluation, however, significant dose differences in the high dose region were identified in the case of NMR evaluation. These findings were contradictory to those provided in [31], where it was shown that significant dose differences were found at low doses. The reason for higher dose differences in the high dose region might be the additional activation of polymerization in irradiated gels and polymer diffusion from the irradiated volume in neighboring areas (peak flattering), since X-rays are used in CT examinations. The impact of X-ray-activated polymer diffusion in gels may be also helpful by explaining similar penumbra values in the TPS and CT evaluated dose profiles. This indicates that NMR evaluation records the state of the art information regarding the gel’s polymerization and corresponding dose distribution in the irradiated volume.

The performed dose profile analysis of the 12 Gy irradiated nMAG gels revealed that 3 mm DTA condition set for gamma analysis using 3%/3mm criteria was fulfilled for both (CT and NMR) read out methods. It should be also noted that the penumbra values in the NMR evaluated gel profiles were in agreement with the EPID-measured penumbra values.

### 2.4. Uncertainty Analysis

The overall uncertainty budget for the gels dosimeters was calculated based on different parameters of uncertainty. These parameters are obtained performing QA measurements of the radiotherapy unit LINAC for a 6 MV FFF photon beam. Assuming that all dose-related uncertainties are independent, the expanded measurement uncertainty for *k* = 2 (95% confidence level) was roughly calculated using following parameters [50]:

(5) $$U\left(D\right)=k{\left({\left(\frac{U_{D}}{\sqrt{3}}\right)}^{2}+{\left(\frac{U_{cal}}{\sqrt{3}}\right)}^{2}+{\left(\frac{U_{B}}{\sqrt{3}}\right)}^{2}+{\left(\frac{U_{E}}{\sqrt{3}}\right)}^{2}+{\left(\frac{U_{F}}{\sqrt{3}}\right)}^{2}+{\left(\frac{U_{\varphi }}{\sqrt{3}}\right)}^{2}+{\left(\frac{U_{T}}{\sqrt{3}}\right)}^{2}\right)}^{\frac{1}{2}}$$

where for the X-ray **CT** case: *U_D_* = ±0.40% is the calibration dose rate uncertainty component, *U_cal_* = ±1.90% is the irradiation equipment calibration component, *U_B_* = ±1.40% is the batch variability, *U_E_* = ±0.50% is the experimental result variations (equipment component), *U_F_* = ±1.20% is the reproducibility component, *U_φ_* = ±1.37% is the calibration curve fit and *U_T_* = ±1.25% is the post-irradiation stability. This gives a combined uncertainty of 1.90% and a total uncertainty of 3.81%;

where for the NMR case: *U_D_* = ±0.40% is the calibration dose rate uncertainty component, *U_cal_* = ±1.90% is the irradiation equipment calibration component, *U_B_* = ±1.40% is the batch variability, *U_E_* = ±0.3% is the experiment result variation (equipment component), *U_F_* = ±1.20% is the reproducibility component, *U_φ_* = ±1.1% is the calibration curve fit and *U_T_* = ±1.25% is the post-irradiation stability. In the case of CT evaluation, the combined uncertainty is 1.83% and the total uncertainty is 3.66%.

The obtained uncertainties were similar to those provided in [51] and were considered being sufficient for the application of the nMAG gels in small field dosimetry. However, taking into account also the relative small gamma pass rate (3%/3mm criteria) values: 90.3% for CT evaluation and 92.7% for NMR, more detailed investigations shall be considered focusing on reproducibility issues and in depth research of post-irradiation processes in irradiated gels.

## 3. Conclusions

The temporal post-irradiation dose response of the nMAG polymer gels irradiated in a small field (<1 cm^2^) geometry with different doses from the interval of 0–24 Gy, has been evaluated using X-ray CT and MRI. A dose sensitivity of 1.76 ± 0.11 HU/Gy was estimated for the dose gels irradiated up to 12 Gy. It was shown that the almost uniform and symmetric CT evaluated dose distribution profiles might be obtained for relatively high doses and after some post-irradiation time. It was found that to get the full scope of the gels’ polymerization and temporal stability, more than 24 h between the irradiation and the evaluation of the gels (as it is recommended now) is needed. However, this post-irradiation time must be not longer than 56 h since, due to gelation and post-irradiative polymerization processes, gel shrinkage causing mass density variations is possible. A gamma passing rate of 90.3% estimated for the CT evaluated 12 Gy irradiated nMAG gels 56 h post-irradiation compared with the data provided by the TPS applying a 3%/3 mm evaluation criteria was the most appropriate among all of the evaluated data, however not optimal. A gamma passing rate of 92.7% for the 3%/3mm criteria was estimated for the NMR evaluated 12 Gy irradiated nMAG gels 56 h post-irradiation. Both of the gamma passing rates were far away from 97%, which is required for an accurate dosimeter. Performed investigation has shown potential for the application of nMAG polymer gels in small field dosimetry, but also indicated limiting gaps which are related to relatively high dose uncertainties: 3.81% for CT evaluation and 3.66% for NMR evaluation. It was shown that a reduction in dose uncertainties is possible considering a more detailed investigation with a focus on post-irradiative polymerization/gelation processes in gels, solving reproducibility issues and optimizing the post-irradiation time frame for the gels’ evaluation.

## 4. Materials and Methods

### 4.1. Samples Preparation

The nMAG gel dosimeters were fabricated in a normal oxygen environment. The 8% gelatin (300 Bloom Type A, Sigma-Aldrich, St. Louis, MI, USA) was mixed with 84% de-ionized water and continuously stirred on the hot-plate magnetic stirrer Heidolph MR (300–500 rpm) at approximately 45 °C until the gel was completely dissolved and a clear solution was obtained. The solution was cooled to 32 °C then the 8% methacrylic acid (MAA, 99%, Sigma-Aldrich) monomer was added and continuously stirred for 25 min until the monomer was completely dissolved. A small amount (10 mM) of Tetrakis (THPC, (hydroxymethyl) phosphonium chloride) was added to the polymer gel as an oxygen scavenger under continuous stirring for 5 min. The manufactured gels were poured into plastic 100 mL containers (H = 6.5 cm, D = 3.5) to have enough space for gel polymerization. Gel-filled containers were tightly closed and stored in a cool dark place for 36 h for solidification.

### 4.2. Irradiation

**Figure 11:**
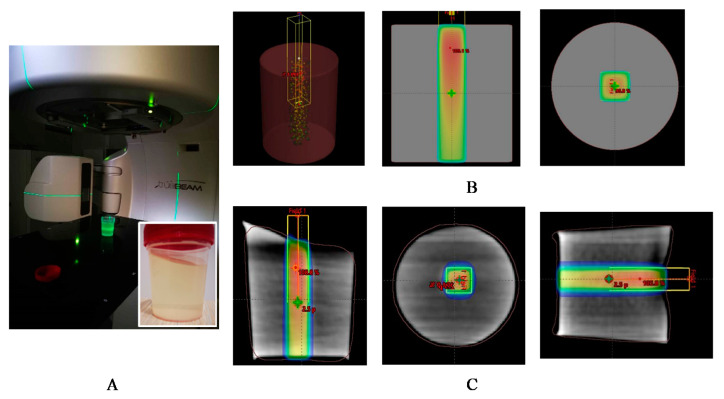
Irradiation of gel samples: (**A**) irradiation set up for small field irradiation of samples with a picture of freshly prepared gel-filled container before sealing; (**B**) small field beam irradiation plan calculated for water phantom by TPS Eclipse; and (**C**) small field beam irradiation plan calculated for real (gel) phantom with indicated dose distributions.

Irradiation of the samples was performed in a linear accelerator (Varian Truebeam STx) using a 6 MV flattening filter-free (FFF) photon beam (Figure 11). Doses from the interval of 6–24 Gy with a maximum dose rate of 1400 MU/min were delivered to the target. An irradiation field of 1 cm^2^ was formed, keeping an SSD of 100 cm. Dose normalization was determined at 2.5 cm depth. MU calculation was performed by Eclipse 15.6 TPS on a virtually created water phantom to accurately estimate the dose at the reference point. Examples of virtual treatment plans calculated using the analytical anisotropic algorithm (AAA) of Eclipse 15.6 are provided in Figure 11B,C for the water phantom and for the real gel phantom, correspondingly. It should be noted that the samples were irradiated from the bottom side. Each sample was labeled according to the delivered irradiation dose.

### 4.3. Readouts

X-ray CT readouts were performed immediately after irradiation of the gel samples and 56, 240 and 2300 h post-irradiation. A selection of “irradiation–scanning” time intervals was performed considering the post-irradiation polymerization dynamics and post-irradiation gelation processes and was based on a literature analysis [45,49] and personal findings of other papers’ authors.

**Figure 12:**
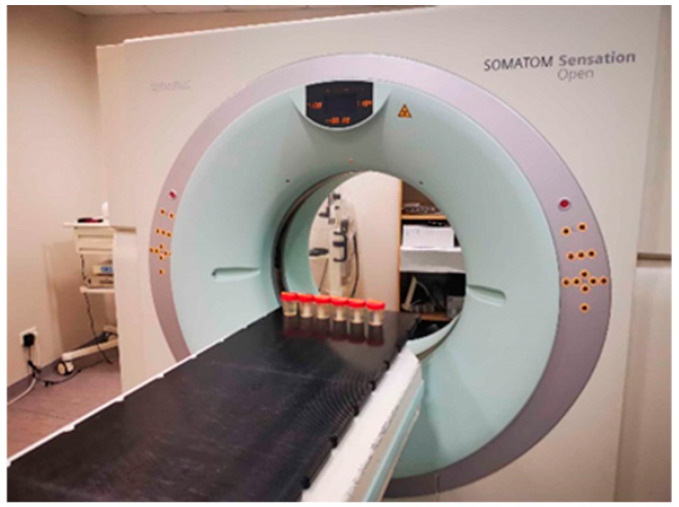
X-ray CT scanning set up for irradiated samples.

A Siemens Somatom Sensation Open CT scanner was used for CT imaging (Figure 12). The CT scanning parameters were as follows: tube voltage of 140 kVp, tube current of 320 mAs and slice thickness of 1.5 mm. Hounsfield units were used as a parameter when evaluating the dose response and dose distribution in the volume.

**Figure 13:**
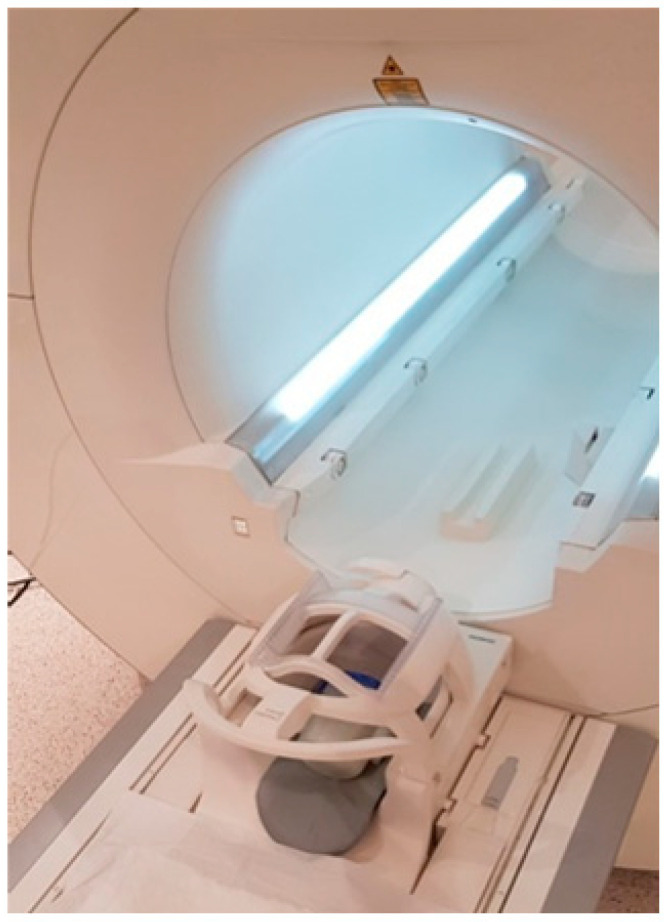
NMR scanning set up with a head coil used for placement of samples.

We used a 1.5T MRI unit Siemens Magnetom Aera to read the polymer gels after irradiation (Figure 13). Vials with the irradiated gel were inserted in a dedicated holder within the head coil. The T2 weighted base imaging sequence was selected for scanning. The scanning parameters were as follows: repetition time 4000 ms, time echo sequences of 92 ms (TE1) and 142 ms (TE2), slice thickness of 5 mm and a voxel size of 0.5 × 0.5 × 0.5 mm.

Since the CT and TPS systems were linked together, the alignment of the samples for CT scanning was realized, creating virtual reference points and using an LAP laser system for the adjustment of the different coordinates. Physical markers were used for adjusting the MRI scanning coordinates for the samples.

## Figures and Tables

**Table 1:** Gamma evaluation results of experimentally (CT read out) obtained dose profiles.

Data Source	Mean Gamma	Lower 95%	Upper 95%
X axis TPS	0.474	0.404	0.557
X axis EPID	0.485	0.413	0.570
Y axis TPS	0.473	0.402	0.555
Y axis EPID	0.512	0.469	0.650
nMAG	0.524	0.475	0.674

**Table 2:** Gamma evaluation results of experimentally (NMR evaluation) obtained dose profiles.

Data Source	Mean Gamma	Lower 95%	Upper 95%
X axis TPS	0.474	0.404	0.557
X axis EPID	0.485	0.413	0.570
Y axis TPS	0.473	0.402	0.555
Y axis EPID	0.512	0.469	0.650
nMAG	0.511	0.455	0.644

**Table 3:** The penumbra and FWHM in EPID and polymer gel-measured dose profiles compared with values provided by TPS for 1 cm^2^ irradiated field size (transverse plane) *.

	CT					NMR				
	FWHM, mm	RPD, %	P(R), mm	P(L), mm	RPD_av_, %	FWHM, mm	RPD, %	P(R), mm	P(L), mm	RPD_av_, %
TPSx	10.01	0	2.0	2.1		10.01	0	1.7	2.0	
TPSy	10.01	0	2.0	2.1		10.01	0	1.7	2.0	
EPIDx	10.02	0	1.6	1.5	27.8	10.02	0	1.8	1.7	5.6
EPIDy	10.01	0	3.0	3.0	37.9	9.30	7.1	3.0	3.0	39.1
nMAG	9.50	5.2	2.3	2.3	11.5	9.50	5.1	3.1	3.0	39.2

* P(L): left penumbra, P(R): right penumbra, FWHM: full width at half maximum, RPD: relative percentage difference (calculated comparing TPS and experimental dose profiles).

## Data Availability

Not applicable.
